# Supplementary material for: A simple and efficient cloning system for CRISPR/Cas9-mediated genome editing in rice
Source: PeerJ. 2020 Jan 29;8:e8491. doi: 10.7717/peerj.8491 (PMC6995270; doi:10.7717/peerj.8491)
Supplement: Supplemental Information 1 [file peerj-08-8491-s001.pdf]

Supplementary Table 1:

Primers used in this study.

Primer	Sequence	Usage
OJD387	aagcaggcttaaagcttAAGGGATCTTTAAACATACGAAC	Constructing donor vectors
OJD388	aaagctgggtaggatccAAAAAAAAGCACCGACTCG	
OJD389	aagcaggcttaaagcttATTTTTTCCTGTAGTTTTCCC	
OJG109	A*gatatc*GAGAGGGATGGGGgttttagagctatgctgaaa	
OJG110	CCCCATCCCTCTC*gatatc*Ttgccacggatcatctgcac	
OJG112	CCCCATCCCTCTC*gatatc*Tggcagccaagccagcaccc	
OJG645	gtgctggcttggctgccGGTCTCTgttttagagctagaaatag	Amplifying the cassette of sgRNA-OsU6a
OJG646	CTATTTCTAGCTCTAAAACAGAGACCGGCAG CCAAGCCAGCAC	
OJK121	ag*ggtctc*ATAACGcAGACCGGCACAC	Removing BsaI sites of pH-Ubi-cas9-7
OJK122	ag*ggtctc*AGGAAAcAGACCTTTTCGACCTT	
OJD383	ACGTGCGCCGGGTTGATGCTttgccacggatcatctgcac	Constructing PJD392
OJD384	AGCATCAACCCGGCGCACGTgttttagagctatgctgaaa	
OJG521	GAAGCGAGCGTACCGGGGAGgttttagagctagaaatag	Constructing PJF645
OJG522	CTCCCCGGTACGCTCGCTTCggcagccaagccagcaccc	
OJH307	ag*ggtctc*AGGCAagtagtatacgtccagctctgttttagagctatgc	Constructing PJF943
OJH308	ag*ggtctc*ACAAACAAATATAAGAGATTCTC ACggcagccaagccagc	
OJI541	GAGATTTTTCAGGATCTTGCT	Genotyping analysis of PJF943
OJI542	AACATAGTATATTGGGCAAGAGG	
OJG723	CACCGCCATCCTCGACAAGCTC	Genotyping analysis of PJD392
OJG724	ATGGTGACACTAACCGTCGTCT	
OJG581	CAATCCGCCGCGCTGGTGT	Genotyping analysis of PJF645
OJG582	AGATGTCCGGGCGCATGTTG	
OJP051	GGGGACAAGTTTGTACAAAAAAGCAGGCTTA	Universal primers
OJP052	GGGGACCACTTTGTACAAGAAAGCTGGGTA	
OJP001	TCGCGTTAACGCTAGCATGGATCTC	Universal primers
OJP002	GTAACATCAGAGATTTTGAGACAC	
