# Supplementary material for: A simple and efficient cloning system for CRISPR/Cas9-mediated genome editing in rice
Source: PeerJ. 2020 Jan 29;8:e8491. doi: 10.7717/peerj.8491 (PMC6995270; doi:10.7717/peerj.8491)
Supplement: Supplemental Information 2 [file peerj-08-8491-s002.pdf]

Supplementary Table 2:

Summary of detected mutations in T0 transgenic plants of each expression clone.

<table>
<tr><th>Target</th><th>NO. of T0 Plants</th><th>NO. of Homozygous Mutants</th><th>NO. of Biallelic Mutants</th><th>NO. of Heterozygous Mutants</th></tr>
<tr><td>OsCCD8</td><td>73</td><td>22</td><td>13</td><td>15</td></tr>
<tr><td>OsDWARF14</td><td>69</td><td>14</td><td>22</td><td>19</td></tr>
<tr><td>OsRCD1 Target 1</td><td rowspan="2">92</td><td>43</td><td>18</td><td>21</td></tr>
<tr><td>OsRCD1 Target 2</td><td>31</td><td>17</td><td>24</td></tr>
</table>
