# Supplementary figures and images for: A simple and efficient cloning system for CRISPR/Cas9-mediated genome editing in rice
Source: PeerJ. 2020 Jan 29;8:e8491. doi: 10.7717/peerj.8491 (PMC6995270; doi:10.7717/peerj.8491)

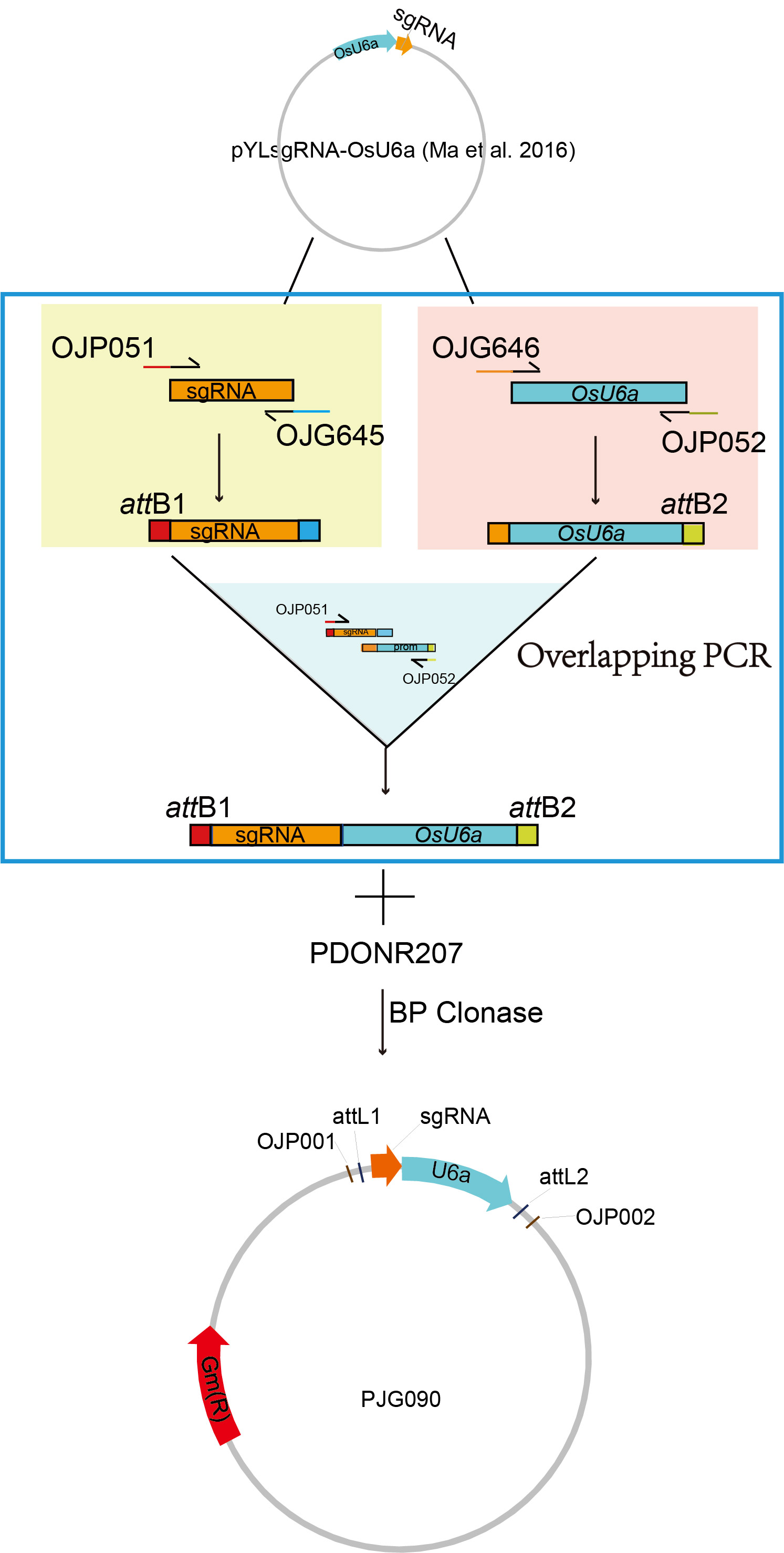

Supplement: Supplemental Information 3 — Overlapping PCR was performed to produce the sequence of sgRNA–OsU6a promoter. The PCR product with attB adaptors was then introduced into pDONR207 with a BP reaction, producing the donor vector named PJG090, which is used as the template for PCR amplification of two spacers. [file peerj-08-8491-s003.jpg]

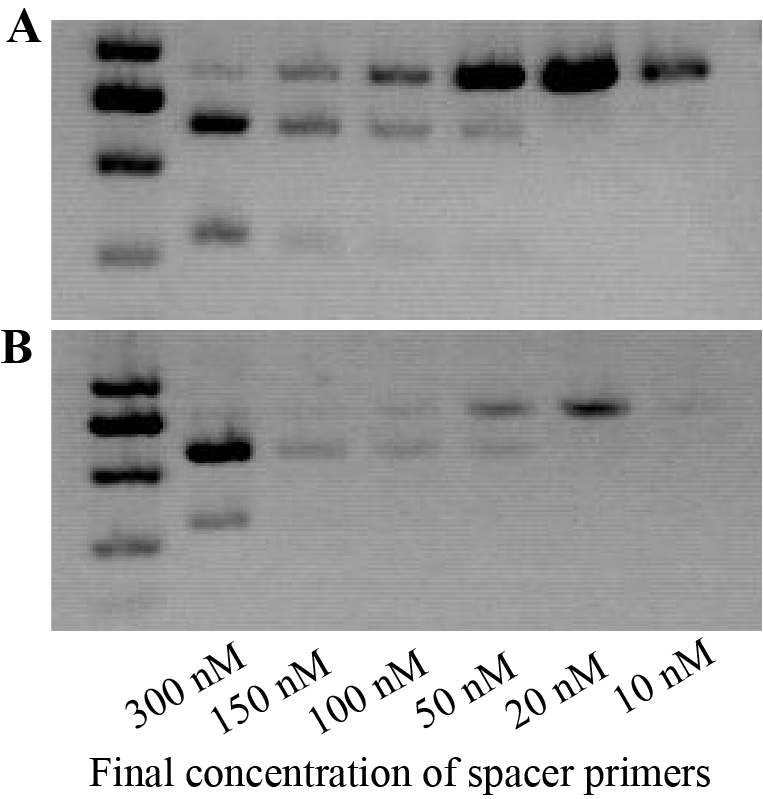

Supplement: Supplemental Information 4 — (A) PJF997 digested by EcoRV was used as the template. OJD383 and OJD384 was used as spacer primers. (B) PJF999 digested by EcoRV was used as the template. OJG521 and OJG522 was used as spacer primers. Multiplex PCR reactions were set up with about 0.1 ng/ul of digested PJF997, universal primers (OJP001 and OJP002, 300 nm each) and spacer primers. To optimize the multiplex PCR, gradient concentration of spacer primers were used. [file peerj-08-8491-s004.jpg]
